# Factors associated with delay in care–seeking for fatal neonatal illness in the Sylhet district of Bangladesh: results from a verbal and social autopsy study

**DOI:** 10.7189/jogh.06.010605

**Published:** 2016-06

**Authors:** Bareng AS Nonyane, Narjis Kazmi, Alain K Koffi, Nazma Begum, Salahuddin Ahmed, Abdullah H Baqui, Henry D Kalter

**Affiliations:** 1 Department of International Health, Johns Hopkins Bloomberg School of Public Health, Baltimore, MD, USA; 2 International Center for Maternal and Newborn Health, Johns Hopkins Bloomberg School of Public Health, Baltimore, MD, USA

## Abstract

**Background:**

We conducted a social and verbal autopsy study to determine cultural–, social– and health system–related factors that were associated with the delay in formal care seeking in Sylhet district, Bangladesh.

**Methods:**

Verbal and social autopsy interviews were conducted with mothers who experienced a neonatal death between October 2007 and May 2011. We fitted a semi–parametric regression model of the cumulative incidence of seeking formal care first, accounting for competing events of death or seeking informal care first.

**Results:**

Three hundred and thirty–one neonatal deaths were included in the analysis and of these, 91(27.5%) sought formal care first; 26 (7.9%) sought informal care first; 59 (17.8%) sought informal care only, and 155 (46.8%) did not seek any type of care. There was lower cumulative incidence of seeking formal care first for preterm neonates (sub–hazard ratio SHR 0.61, *P* = 0.025), and those who delivered at home (SHR 0.52, *P* = 0.010); and higher cumulative incidence for those who reported less than normal activity (SHR 1.95, *P* = 0.048). The main barriers to seeking formal care reported by 165 mothers included cost (n = 98, 59.4%), believing the neonate was going to die anyway (n = 29, 17.7%), and believing traditional care was more appropriate (n = 26, 15.8%).

**Conclusions:**

The majority of neonates died before formal care could be sought, but formal care was more likely to be sought than informal care. There were economic and social belief barriers to care–seeking. There is a need for programs that educate caregivers about well–recognized danger signs requiring timely care–seeking, particularly for preterm neonates and those who deliver at home.

Recent estimates show that 6.3 million children died in 2013, which is a decline from 12.7 million in 1990 [1]. Of these 6.3 million, 2.8 million babies died in the neonatal period (within 28 days of birth) making up 44% of all under 5 deaths, a trend that has been observed over the last two to three decades [2–4]. Most of the under–5 deaths occur in developing countries where the estimated rate in 2012 was 53 per 1000 live births (90% uncertainty bound 51,56) compared to 6 (6,7) per 1000 live births in developed countries [5]. Most of the deaths in developing countries occur due to preventable causes for which care could be made available by targeted interventions [6]. Liu et al [7] estimated that in 2013, 2.761 million (44%) of the under–5 deaths were in the neonatal period, and among those 0.42 million neonates died of sepsis. Appropriate and timely care–seeking for infections could substantially improve neonatal survival. A systematic review of care–seeking behaviors for neonatal and childhood illnesses in low and middle–income countries showed that levels of care–seeking vary considerably by geographic region, with levels in South Asia being particularly low [8].

## Delays in care–seeking and constraining factors

Care–seeking by itself is important, but even more important is the ability to reach out to available qualified health care providers as soon as the illness signs are recognized. The 3–delays model for care–seeking for maternal illnesses was developed by Thadeus and Maine (1994) [9] and has been applied to characterize the delay in seeking care for childhood illnesses [10–14].This model identifies the delay in 1) recognizing danger signs and deciding to seek care, 2) time to get to the health facility and 3) receiving adequate and appropriate care after reaching the facility.

Traditional beliefs and cultural practices have been shown to influence decision–making and the time to seeking care [15–19]. A specific example is that in Rajasthan, India, care–seeking for sick neonates was found to be less than that for older infants and children, mainly due to caretakers believing that even qualified providers lack the expertise to treat newborns [16–18]. Qualitative research in three South Asian countries (Bangladesh, Nepal and Pakistan) found that local traditions, lack of knowledge about the importance of care–seeking and recognition of danger signs and perceived poor quality of health services were important factors [19]. A focused ethnographic study in India found that despite caretakers recognizing danger signs indicating that their child needed health care, inability to discriminate among the available health care sources and perceived poor quality of health services led them to delay seeking care or to seek care from unqualified providers [20].

Similar results have been found in Sub–Saharan Africa and Latin America. For example, in some cases, caregivers assume that they know what the illness is and treat it at home instead of seeking formal care [12]. Other influences include lack of knowledge about dangerous illness symptoms [21], and various factors that ranged from social and traditional beliefs as well as poor health systems [10,11,22–25].

## Care–seeking in Bangladesh

Bangladesh has a total population of 160 million and 9.6% of the population is under 5 years of age. The Bangladesh Demographic and Health Survey shows that the country had an under–5 mortality rate of 53 per 1000 live births and neonatal mortality rate of 32 per 1000 live births in 2011 [26]. Even though the country has met the Millennium Development Goal (MDG) 4 of reducing child mortality by two–thirds between 1990 and 2015, the neonatal mortality rate is still high and it requires targeted interventions to promote timely care–seeking for neonatal illness.

In a study done in rural Bangladesh to assess the care–seeking patterns for neonatal morbidity, it was seen that although most of the mothers sought outside care, only a small percentage of those who sought care considered going to a qualified provider and that was true even for the neonatal mortalities [27]. A recent study in Mirzapur Bangladesh found that even for older children (1–59 months) caretakers prefer to visit unqualified providers or other sources as compared to a formal provider [28]. Another study from Sylhet Bangladesh showed that preventative or curative care was sought for only 30.9% of preterm newborns from qualified providers [29]. A study by Chowdhury et al. [30] in Matlab, Bangladesh found that 37% of 365 neonates who had a fatal illness had formal care sought for them while the rest either received traditional or no care. The authors highlighted the need to design programs that take into consideration the use of traditional care and formal care in order to promote timely care–seeking.

## Verbal and Social Autopsy studies to study care–seeking behavior

In low– and middle–income countries (LMICs), death registries are often either poorly kept or non–existent and, as a result, verbal autopsies (VAs) are used to help determine the likely cause of death. These are questionnaire instruments that are used to collect reported illness symptoms and information on pregnancy and intra–partum complications, in the case of neonates. The data are then used to determine the likely cause of death using physician assessments or expert algorithms [31–35]. Social autopsies (SAs) are conducted to help determine social and health system factors associated with care–seeking behavior [36,37].

There is very limited literature on comparative studies of those who seek formal care vs those who do not, and the factors associated with that decision [8,38]. In this study, we used data from a verbal and social autopsy (VASA) study in the Sylhet district of Bangladesh. We conducted an exploratory analysis to determine the illness symptoms as well as social and demographic factors that were associated with the delay in seeking formal care for a neonatal illness that led to death. Our analysis accounted for the competing risks of death and seeking informal care before formal care could be sought.

## METHODS

### Setting and participants

The VASA survey data were collected from four unions of Zakiganj sub–district with an estimated population of 102 000; and four unions of Kanaighat sub–district with an estimated population of 100 000 in the Sylhet district of Bangladesh. Initially, data were collected with a 1 year recall period, and this was extended for up to 2.5 years in order to attempt to achieve the desired sample size of up to 500 neonatal deaths. This sample size was determined for estimating the cause of death distribution (the first objective of the study) with 5% precision for the main common causes of death. The current–study’s objective (delay in care–seeking) utilized a subset of these neonatal deaths that fitted the inclusion criteria. Babies born to ever–married mothers of child–bearing age (15 to 49 years old) were included. Deaths that occurred between October 2007 and May 2011 were included. Respondents were selected among participants of other studies on community–based interventions for maternal and newborn care. These were the Healthy Fertility study, which was aimed at improving healthy birth–spacing [39], and the Chlorhexidine trial which was a three–arm trial comparing the effect of umbilical cord cleansing with chlorhexidine once, over a 7–day period, and the control arm which was dry cord–care [39,40].The verbal autopsy questionnaires were administered retrospectively by trained female data collectors. Social autopsy interviews were conducted by trained female interviewers. Mothers who had had multiple deaths were interviewed for each of the deaths separately. The questionnaires used were the World Health Organization’s (WHO) standard verbal autopsy tool [41] and the WHO/UNICEF–supported Child Health Epidemiology Reference Group (CHERG) social autopsy tool [36,42].

### Analysis

The main event of interest was the time to seeking formal care, reported in days since illness onset. Illness onset was defined as the time when the first symptoms were recognized. Formal care in this context was defined as care provided by one of the following: a trained community health worker (CHW), private doctor or NGO/Government health center/post or hospital. Informal care was defined as seeking care from a traditional healer or from a pharmacist/drug seller. Other than the main outcome of seeking formal care, there were other possible events that may have taken place before formal care was sought and they were important to consider because they could have altered the probability of seeking formal care. These were a) death before any care was sought (survival bias) and b) seeking informal care first or only, and they were referred to as *competing risk events* [43]. Failure to account for these competing events (that is treating them as censoring events) may lead to an over–estimation of the incidence of seeking formal care and the effect estimates of the potential predictors. We calculated and plotted the cumulative incidence functions (the probability of an event of type *k* before or up to time *t*) for each of the possible events [44].

We then built a semiparametric regression model to estimate the cumulative incidence of seeking formal care first in the presence of competing events, and we report corresponding sub–hazard ratios (SHRs) with respect to each predictor [45,46]. The effect estimate is referred to as a ‘sub–hazard’ ratio because it pertains to one event among all possible events in any given time point. This model is analogous to the Cox proportional hazards model except that hazard ratios for an event *k (*such as seeking formal care) are calculated conditional on an individual having had no other event up to time *t*. SHRs are interpreted as a reduction or increase in cumulative incidence of an event. Unadjusted regression models were fitted for each potential predictor separately, and the predictors with a *P*–value ≤0.2 were included in the multivariable model. We investigated possible multi–collinearity among some of the predictors and its effect on the interpretation of the results.

A subset of the mothers who had not taken their neonate to a formal health care provider said that they had ‘concerns’ that prevented them from doing so. A subset of those who had sought formal care said they had ‘concerns’ that they had to overcome in doing so. Furthermore, those mothers who reported that their neonates died immediately were never asked about any concerns they had. These concerns were potential barriers to care–seeking and since they were not applicable or answered by all respondents, they were not included in the main regression analysis and only summary statistics of these are given.

### Inclusion/exclusion

In order to assess care–seeking from home, only participants whose baby was either born at home, or left the delivery facility alive, were included in the analysis.

### Potential predictors

We considered the following classes of predictors as shown in **Table 1**: neonate’s demographic factors, neonatal care variables, illness symptoms, mother’s/father’s factors, household factors, social and health system factors. We used the WHO’s Integrated Management of Childhood Illnesses (IMCI) severity grading for the first symptoms they observed. For the illness symptoms that were in the VA instrument but not in the IMCI, two physician authors (HDK, AKK) assigned symptoms as severe (requiring referral to higher level formal care) or possibly severe (requiring formal health care). The listing of the symptoms and their severity scoring are given in **Online Supplementary Document(Online Supplementary Document).**

**Table 1:** Potential predictors for care–seeking behavior

Factor	Variable
Neonate’s demographic factors	• Age of neonate at illness onset (days) • Gender
Neonates care, illness symptoms/conditions	• Neonate ever breastfed? • Whether the newborn received any liquids or solids other than breast milk (exclusive breastfeeding); • Severity of the observed first symptoms for which care was reportedly sought • Birth size of the baby • Whether the neonate was preterm or not • Whether the baby had any malformation at birth • Mother/care–giver’s perception of the status at illness onset: – feeding status (feeding normally, poorly or not at all) – activity (normally active, less active than normal or not moving)
Mother/father’s factors	• Mother’s age • Mother’s education • Father’s education • Whether the mother sought any antenatal care at a health provider • Whether mother had any pre–pregnancy medical condition
Household factors	• Whether mother was the household breadwinner • Where the mother stayed during the last days of her pregnancy
Specific barriers	• Any specific concerns or problems caregiver had: Thought baby not sick enough; no one available to go with caregiver; too much time from regular duties; someone else had to decide; too far to travel; no transportation available; cost; not satisfied with available health care; problem required traditional care; thought too sick to travel; thought child will die anyway; it was late at night; no transport/provider; other
Health system factors	• Delivery place • Time in minutes to usual health provider

### Ethical considerations

The VASA study was approved by the Institutional Review Boards of the Johns Hopkins University and the Bangladesh Medical Research Council. All respondents provided informed consent before being interviewed.

## RESULTS

A total of 378 death records were available for analysis. Of these, 46 died at the facility in which they were born and thus, per inclusion/exclusion criteria, were excluded from the analysis; one had an unknown place of birth; and the rest (n = 331) were included in the analysis. The 331 comprised 307 babies born at home and 24 born at a facility and discharged alive. Furthermore, 12 of these mothers had given interviews about 2 deaths each. The recall period was 1 year in the initial study plan and under this criterion 72.5% (240/331) of the verbal autopsies were conducted. As the recall period was extended to 2.5 years in order to increase sample size, an additional 27.5% (91/331) VAs were conducted.

**Figure 1:**
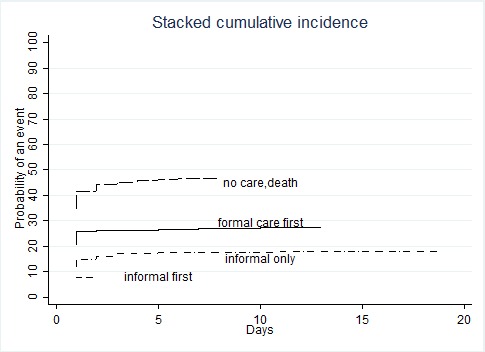
Cumulative incidence plots.

**Table 2** shows some basic characteristics of these 331 participants and the types of care sought for them. The outcome of interest was seeking formal care first; and this was done for 91 (27.5%) neonates while 26 (7.9%) sought informal care before seeking formal care. Informal care only was sought for 59 (17.8%) neonates and 155 (46.8%) died before any care could be sought for them. The cumulative incidence functions for each of these competing events are given in **Figure 1**. These show that dying without any care sought was the most likely outcome, followed by seeking formal care first and seeking informal care only, while seeking informal care first was the least likely event.

**Table 2:** Baseline characteristics of participants and care–seeking behavior

Variable	N = 331	%	Median (interquartile range)
Neonate’s gender = female	147	44.4	
Age (days) at illness onset			0 (0,2)
Place of birth = hospital/facility	25	7.5	
Formal care sought first	91	27.5	
Informal care sought first	26	7.9	
Informal care only sought	59	17.8	
No care sought	155	46.8	
Median time (day of illness) to first seeking formal care			1 (1,2)
Median time (days) from illness onset to death			1 (0,2)
Median time(days) to informal care–seeking			1 (1,2)

The results of the unadjusted regression analysis for each potential predictor are given in **Table 3**, and the predictors with a *P*–value ≤0.2 were included in the multivariable model. The results from the multivariable model (**Table 4**) show that there was a lower cumulative incidence of seeking care for preterm vs full–term neonates (SHR = 0.61, *P* = 0.025) and those who delivered at home (SHR = 0.52, *P* < 0.010). Those who reported less than normal activity in a neonate were more likely to seek formal care than those who reported normal activity (SHR = 1.95, *P* = 0.025).

**Table 3:** Characteristics of the sample and results of unadjusted regressions for the time in days to first instance of seeking formal care

Variable	N = 331	Sought formal care first (n, %)		Sub–hazard ratio for time to first seeking formal care	*P*–value
**Age at illness onset–days:**					
0	216	58 (26.9)		1	
1–3	26	8 (30.8)		1.2	0.64
3+	87	24 (27.6)		1.1	0.8
Missing	2				
**Gender:**					
Male	184	57 (30.9)		1	
Female	147	34 (23.1)		0.7	0.09
**Newborn ever breastfed:**					
No	170	38 (22.4)		1	
Yes	160	53 (33.3)		1.5	0.03
Don’t know	1				
**Were first symptoms observed severe/possibly severe?**					
No	19	1 (20.3)		1	
Yes	267	78 (29.2)		2.74	0.15
**Birth size:**					
Very small/smaller than usual	214	58 (27.1)		1	
Average/larger than usual	117	33 (28.2)		0.99	0.96
**Preterm birth:**					
No	227	69 (30.4)		1	
Yes	103	22 (21.4)		0.66	0.08
Don’t know	1				
**Malformation at birth:**					
No	325	89 (27.4)		1	
Yes	6	2 (33.3)		1.2	0.81
**Feeding status at illness onset:**					
Normal	43	11 (25.6)		1	
Poorly	109	41 (37.6)		1.5	0.21
Not at all	178	39 (21.9)		0.83	0.56
**Activity status at illness onset:**					
Normally active	53	10 (18.9)		1	
Less active than normal	182	64 (35.2)		1.9	0.04
Not moving	94	17 (18.1)		0.95	0.83
Missing	2	0			
**Mother’s age:**					
18–20years	52	18 (34.6)		1	
21–25years	110	36 (32.7)		0.88	0.61
26–30years	97	18 (18.6)		0.47	0.02
>30years	72	19 (26.3)		0.72	0.27
**Mother’s education in years:**					
0 years	113	26 (23.0)		1	
1–5 years	109	28 (25.7)		1.13	0.63
6–12 years	109	37 (33.9)		1.53	0.07
**Any antenatal care for mother:**					
No	142	90 (27.4)		1	
Yes	189	1 (33.3)		1.22	0.83
**Mother had any pre–existing medical conditions:**					
No	312	85 (27.2)		1	
Yes	19	6 (31.6		1.19	0.66
**Breadwinner:**					
Child’s father	297	81 (27.3)		1	
Other	34	10 (29.4)		1.10	0.75
**Place stayed during last days of pregnancy/during fatal illness:**					
Own/current	303	83 (27.4)		1	
Parent’s	25	7 (28.0)		1.02	0.96
Missing	3				
**Mother able to turn to others for help?**					
No	187	46 (24.6)		1	
Yes	144	45 (31.3)		1.30	0.17
**Mother/her family ever been denied any community service:**					
No	325	89 (27.4)		1	
Yes	6	2 (33.3)		1.24	0.73
**Time to usual provider in minutes:**					
<25	187	46 (24.6)		1	
≥25	144	45 (31.5)		1.28	0.19
Don’t know	1				
**Delivery place:**					
Facility	25	15 (60.0)		1	
Home	306	76 (24.8)		0.38	<0.001

**Table 4:** Results from the multivariable regression of time to first instance of seeking formal care

Predictor	Sub–hazard ratio	*P*–value	95% CI lower limit	95% CI upper limit
Gender (male is reference)	0.72	0.094	0.46	1.06
Neonate ever breastfed by anyone? (no is reference)	1.45	0.106	0.92	2.28
Any severe symptom seen (no is reference)	2.54	0.181	0.65	9.92
Preterm (no is reference)	0.66	0.025	0.39	0.94
Activity at illness onset = less than normal (normal is reference)	1.95	0.048	1.01	3.78
Activity at illness onset = not moving (normal is reference)	1.12	0.796	0.48	2.59
Mother's age 21–25 y (18–20 y is reference)	1.07	0.759	0.67	1.73
Mother's age 26–30 y (18–20 y is reference)	0.56	0.057	0.31	1.02
Mother's age >30 (18–20 reference)	0.97	0.947	0.52	1.86
Mother's education 1–5 y (0 is reference)	1.19	0.486	0.72	1.99
Mother's education 6–12 y (0 is reference)	1.33	0.272	0.80	2.20
Mother could turn to others for help (no is reference)	1.14	0.486	0.79	1.64
Time to usual provider in minutes ≥25 (<25 is reference)	1.22	0.269	0.86	1.74
Delivery place = home (hospital/facility is reference)	0.52	0.010	0.32	0.85

CI – confidence interval, y – years

**Table 5** shows the summary data on the barriers to seeking care raised by respondents. A subset of 165 mothers reported having these concerns or barriers, and among those, 39 had taken their neonates to seek formal care despite the barriers. The most common barrier was cost, which was raised by 98 (60%) of the mothers, 67 of whom had not sought formal care. The next common barriers were: thinking that the baby would die anyway (n = 29, 18%), thinking the baby needed traditional care (n = 26, 16%), being too late at night to travel (n = 19, 12%), and the distance to the formal care facility (n = 18, 11%).

**Table 5:** Specific concerns/problems that were barriers to formal care–seeking–reported by mothers

	Total (N = 165)	Total N = 39 (of 165) sought formal care	Total N = 126 (of 165) who did not seek formal care	
	**n (%)**	**n (%)**	**n (%)**	
**Mother’s specific concern – thought not sick enough:**				
No	151 (91.5)	39 (100)	112 (88.9)	
Yes	14 (8.5)	0	14 (11.1)	
**Mother’s specific concern – no one available to go with her:**				
No	157 (95.2)	39 (100)	118 (93.7)	
Yes	8 (4.8)	0	8	(6.3)
**Mother’s specific concern – cost:**				
No	67 (40.6)	6 (15.4)	61 (48.4)	
Yes	98 (59.4)	33 (84.6)	65 (51.6)	
**Mother’s specific concern – too much time from regular duties:**				
No	148 (89.7)	38 (97.4)	110 (87.3)	
Yes	17 (10.3)	1 (2.6)	16 (12.7)	
Mother’s specific concern – someone else’s decision:				
No	153 (92.7)	37 (94.9)	116 (92.1)	
Yes	12 (7.3)	2 (5.13)	10 (7.9)	
**Mother’s specific concern – too far to travel:**				
No	147 (89.1)	36 (92.3)	111 (88.1)	
Yes	18 (10.9)	3 (7.7)	15 (11.9)	
**Mother’s specific concern – no transport:**				
No	161 (97.6)	38 (97.4)	123 (97.6)	
Yes	4 (2.4)	1 (2.6)	3 (2.4)	
**Mother’s specific concern – thought needed traditional care:**				
No	139 (84.2)	38 (97.4)	101 (80.2)	
Yes	26 (15.8)	1 (2.6)	25 (19.8)	
**Mother’s specific concern – too sick to travel:**				
No	151 (91.5)	38 (97.4)	113 (89.7)	
Yes	14 (8.5)	1 (2.6)	13 (10.3)	
**Mother’s specific concern – thought would die anyway**				
No	136 (82.4)	36 (92.3)	100 (79.4)	
Yes	29 (17.6)	3 (7.7)	26 (20.6)	
**Mother’s specific concern – late at night:**				
No	146 (88.5)	37 (94.9)	109 (86.5)	
Yes	19 (11.5)	2 (5.13)	17 (13.5)	
**Mother’s specific concern – not satisfied with service at formal health facility:**				
No	165 (100)	39 (100)	126 (100)	
Yes	0	0	0	
**Mother’s specific concern – other reasons:**				
No	157 (95.2)	38 (97.4)	119 (94.4)	
Yes	8 (4.9)	1 (2.6)	7 (5.6)	

## DISCUSSION

Care–seeking for neonatal and child illnesses in resource–limited settings is low. In this study, we aimed to determine factors associated with care–seeking behavior for fatal neonatal illness in Sylhet, Bangladesh, using data from verbal and social autopsy questionnaires. Our main outcome of interest was time to the first instance of seeking formal care. In order to conduct such an analysis, it was crucial to condition, at each time point during follow–up, on the neonate having survived a competing risk of death or seeking informal care first. Thus a competing risk time to event model was used.

In this Bangladesh cohort, the cumulative incidence analysis showed that neonates were more likely to die before any care could be sought, but that if care was sought, it was more likely to be formal vs informal care. Unadjusted analyses indicate that, formal care was sought first for only 27.5% of the sick neonates, and 47% never sought any care outside of the home. These results are similar to findings from other studies in rural Bangladesh with a level of care–seeking for fatal neonatal illnesses ranging from 35 to 37% [29,30].

Neonates whose activity level was reportedly less than normal had formal care sought first at a significantly higher rate than for those who were reported to have normal activity, while those who were reportedly not moving were neither more nor less likely to have formal care sought for them. This is evidence that perception of illness played a role in decision–making, and suggests that those who were reportedly not moving were thought to be less likely to survive even if care was sought. On the other hand, for those who were moving less than normal there was hope that they could survive if formal care was sought first. This corroborates the finding from other studies conducted in India and Ghana that found that lethargy is one of the few signs of neonatal illness that mothers both recognize and understand to indicate the need for formal health care [15–17].These studies distinguished such signs from others that mothers recognized but took to mean that traditional or no care was required and other illness signs that were poorly recognized. Thus, the current study adds to the evidence suggesting that focusing health messages on a few well–recognized and intrinsically motivating illness signs may be more effective in increasing formal health care–seeking for sick neonates than a strategy that urges care–seeking for all danger signs.

Preterm babies had care sought for them at a lower rate than full–term neonates. We found that among the subset that reported barriers to seeking formal care, 24% of the mothers who had preterm babies thought the babies would die anyway while only 14% of the mothers with full–term neonates felt that way. Thus, even though it is not possible to adjust for the reported barriers in the main model, it may be that being preterm led the mothers to think that there was no hope of survival.

We investigated whether those who were born at a health facility survived longer before illness onset and whether care–seeking decisions were different for them. The numbers that can be used to make any such inference were small. Of the total 331 participants, 25 were born at a facility. Of those 25, 15 (63%) reported that illness began on day 1 of life, which is comparable to 66% of the homebirths. Furthermore, 17 (71%) of the facility births sought formal care on the same day as illness onset, which is also comparable to the 228 (75%) of home–births. Hence, we conclude that the small subset of facility births were not different in terms of their survival before illness onset, nor by the care–seeking decisions made for them.

Neonates who were delivered at home were less likely to have care sought for them. This was an expected finding because mothers who are less likely to seek care at facilities are also less likely to deliver there. In this Bangladesh cohort, the reported barriers to seeking care have also been found in similar low–income settings [10,11,22–25]. The main ones were the cost, having no hope of survival or believing that traditional care was more appropriate for some illnesses.

### Strengths and limitations

The main strength of our study is that it was a comparative analysis of those caregivers who did vs those who did not seek formal care, accounting for competing risks of death or seeking informal care first. It thus allowed for a more definitive determination of the factors that constrain prompt formal health care–seeking fatally ill neonates. Data were collected by highly–trained and skilled female verbal autopsy interviewers working for the HFS and CHX studies as well as for the social autopsy component. Furthermore, this was a community–based study that included both home and hospital births, and thus it did not suffer selection bias that would occur if only hospital births had been included.

A limitation to our study is that there may have been bias in self–reported time to seeking care (days/h) given that the recall period was up to 2.5 years for some of the respondents.

Not all mothers responded to the questions of the specific barriers that prevented them from seeking care or that they had to overcome. Thus it was not possible to include responses to these barriers in the main multivariable model. However, these reported barriers have also been found to apply in other similar settings.

The time to care–seeking data was not presented in hours for all types of events and thus we were not able to fully describe the pattern of events for the first day of life which is a crucial time when most deaths and actions are taken on. Time in hours was only asked of those who sought care at a formal provider and thus we could not give a comparative analysis of the events.

## CONCLUSION

Our analysis has shown that in this rural Bangladesh cohort, the majority of the neonates died before formal care could be sought for them, but when care was sought it was more likely to be formal vs informal care. There were economic and social belief barriers that delayed or prevented care–seeking. There is a need for programs that address such barriers and educate caregivers about danger signs that require formal health care and the importance of timely care–seeking, particularly for preterm neonates and those who deliver at home.
