# Supplementary material for: Factors associated with delay in care–seeking for fatal neonatal illness in the Sylhet district of Bangladesh: results from a verbal and social autopsy study
Source: J Glob Health. 2016 May 1;6(1):010605. doi: 10.7189/jogh.06.010605 (PMC4920004; doi:10.7189/jogh.06.010605)
Supplement: Online Supplementary Document [file jogh-06-010605-s001.pdf]

Online Supplementary Document

Nonyane et al. Factors associated with delay in care–seeking for fatal neonatal illness in the Sylhet district of Bangladesh: results from a verbal and social autopsy study

JoGH 2016;6:010605

Severity of symptoms grading

1. **Possibly severe illness signs:**
   - Boils
   - More frequent loose or liquid stools than usual
   - Injury in head / neck
   - Did not cry
   - Smaller than normal
   - Umbilical redness

2. **Severe illness signs:**
   - Fast breathing
   - Chest indrawing
   - Stopped being able to breastfeed
   - Less movement
   - Grunting
   - Fever
   - Body cold
   - Spasms or convulsions
   - Unresponsive or unconscious
   - Vomiting
   - Did not breath immediately after birth
   - Difficulty from birth, no route to passing stool or cleft palate
   - Not able to open mouth
   - Areas of the skin turned black
   - "Blood in body or mouth/nose" or "bleeding through cord" or "bleeding through anus"
   - Jaundice: "Body/ eyes become yellow"
